# Draft Genome Sequence of Quorum-Sensing and Quorum-Quenching *Pseudomonas aeruginosa* Strain MW3a

**DOI:** 10.1128/genomeA.00258-14

**Published:** 2014-04-17

**Authors:** Kok-Gan Chan, Cheng Siang Wong, Wai-Fong Yin, Xin Yue Chan

**Affiliations:** Division of Genetics and Molecular Biology, Institute of Biological Sciences, Faculty of Science, University of Malaya, Kuala Lumpur, Malaysia

## Abstract

*Pseudomonas aeruginosa* has a broad range of habitation, from aquatic environments to human lungs. The coexistence of quorum-sensing and quorum-quenching activities occurs in *P. aeruginosa* strain MW3a. In this work, we present the draft genome sequence of *P. aeruginosa* MW3a, an interesting bacterium isolated from a marine environment.

## GENOME ANNOUNCEMENT

*Pseudomonas aeruginosa* is classified under the *Gammaproteobacteria* and lives in a broad range of environments, including natural environments, eukaryotic hosts, and man-made products (1–3). *P. aeruginosa* utilizes its quorum-sensing ability to communicate within its community in order to coordinate community behaviors that enhance adaptivity to various environments (4–6). Aside from quorum sensing, *P. aeruginosa* also possesses quorum-quenching mechanisms that enzymatically degrade its quorum-sensing signaling molecules (7). The degradation of its quorum-sensing signaling compound enables the self-regulation of *P. aeruginosa* quorum sensing, while the degraded compound acts as the bacterial growth nutrient (7, 8).

*P. aeruginosa* strain MW3a was isolated from the subsurface level (5 cm beneath sea level) in the Strait of Malacca using KGm medium (9, 10). Preliminary studies have shown that this isolate possesses both quorum-sensing and quorum-quenching abilities. *N*-Dodecanoyl-l-homoserine lactone and *N*-3-oxotetradecanoyl-l-homoserine lactone were detected from the spent supernatant of this isolate by high-resolution mass spectrometry. In addition, *N*-acyl-homoserine lactone (AHL) degradation was observed by rapid-resolution liquid chromatography, with a preference on AHL with a 3-oxo group substitution.

Total genomic DNA of *P. aeruginosa* strain MW3a was extracted and purified with the QIAamp DNA minikit (Qiagen, Germany). Subsequently, the purified genomic DNA was subjected to whole-genome shotgun sequencing on an Illumina HiSeq (Illumina, Inc., USA) platform. Quality reads were *de novo* assembled with the CLC Genomics Workbench 6.0.5 (CLC bio, Denmark). Gene prediction was performed with the prokaryotic gene prediction algorithm Prodigal (version 2.60) (11), while rRNAs were predicted with RNAmmer (12). Subsequently, the genome sequence was annotated with BLASTx against the NCBI-nt/nr and UniProt databases (13, 14).

The whole-genome sequencing generated 25,826,420 paired-end reads, with an average length of 101 bp. The filtered reads were *de novo* assembled into 240 contigs with a length of ≥200 bp, and an *N*_50_ of 81.6 kb was generated. The draft genome of *P. aeruginosa* MW3a contains 6,665,300 bases, with an average coverage of 366-fold and a G+C content of 66.29%. The gene prediction resulted in 6,288 open reading frames (ORFs), and a copy each of 5S rRNA, 16S rRNA, and 23S rRNA was identified.

Based on the BLAST result, two AHL-based quorum-sensing homologs were detected from the draft genome of *P. aeruginosa* MW3a. The *rhl* quorum-sensing system, which is responsible for short-chain AHL synthesis, was found in contig 7, while the *las* system, which synthesizes long-chain AHL, was carried in contig 158. On the other hand, the *quiP* and *pvdQ* genes that encode AHL acylase were found in contigs 98 and 107, respectively.

### Nucleotide sequence accession numbers.

This whole-genome shotgun project has been deposited at DDBJ/EMBL/GenBank under the accession no. JAQK00000000. The version described in this paper is the first version, JAQK01000000.
